# Impact of comorbidity on survival in cancer patients receiving immune checkpoint inhibitors

**DOI:** 10.1007/s12094-025-03848-7

**Published:** 2025-01-28

**Authors:** Merih Yalçıner, Satı Coşkun Yazgan, Eda Eylemer Mocan, Beliz Bahar Karaoğlan, Hatice Bölek, Emre Yekedüz, Yüksel Ürün

**Affiliations:** Medical Oncology Department, Faculty of Medicine, School of Medicine, Cebeci Hospital, Ankara University, Dikimevi, 06590 Ankara, Turkey

**Keywords:** Comorbidity, Immunotherapy, Immune checkpoint inhibitors

## Abstract

**Purpose:**

Immunotherapy efficacy in elderly patients with comorbidities and poor performance status is not well understood. More knowledge on this topic is needed to identify subgroups that will benefit from immunotherapy. We aimed to evaluate the effect of comorbidity burden in patients receiving immunotherapy.

**Methods/patients:**

Patients older than 18 years of age and diagnosed with various malignancies, followed up in our tertiary cancer center were screened. Patients treated with immunotherapy were included in this study. We used to Charlson Comorbidity Index (CCI) to evaluate patients’ comorbidity burden. The primary outcome was overall survival (OS). Hazard ratio (HR) with confidence interval (CI) was evaluated in multivariable analysis.

**Results:**

A total number of 197 patients were included. The median age was 62 years. Patients were grouped based on CCI scores: CCI-low (≤ 8) and CCI-high (> 8). One-hundred and seven patients (54.9%) had metastatic disease at the time of diagnosis. Most frequently used immunotherapy agent was nivolumab (*n* = 124, 62.9%), followed by pembrolizumab (*n* = 36, 18.3%). The median OS was shorter in the CCI-high group than in the CCI-low group (10.6 vs. 21.2 months, *p* = 0.002) In multivariable analysis, treatment with anti-CTLA4 (HR: 1.85, 95% CI 1.07–3.20, *p* = 0.028), ECOG performance status (2–4 vs. 0–1) (HR: 2.17; 95% CI 1.25–3.75; *p* = 0.005), and higher CCI scores (CCI-high vs. CCI-low) (HR: 1.97; 95% CI 1.3–3.0; *p* = 0.001) were independently associated with worse OS.

**Conclusions:**

Comorbidity burden and performance status independently predict survival outcomes in immunotherapy-treated cancer patients. Comprehensive comorbidity assessment is essential for optimizing treatment and improving patient outcomes.

**Supplementary Information:**

The online version contains supplementary material available at 10.1007/s12094-025-03848-7.

## Introduction

The cancer treatment landscape has evolved at a great pace since the introduction of immune checkpoint inhibitors (IO), which lead to superior outcomes in many types of cancer. As a result, biological and clinical parameters that may predict response to IO treatment have emerged as crucial research topics. Evolving tumor immunology reveals novel biomarkers as therapeutic targets and response predictors [1–4]. However, even though age is one of the most important risk factors for various types of cancer, our knowledge of immunotherapy efficacy on elderly patients, especially those with lower performance status and comorbidities, remains limited.

Several reasons could be explanatory for this knowledge gap. Elderly patients with severe comorbidities are less likely to meet eligibility criteria for clinical trials, which leads to the underrepresentation of this population. It is well known that age, comorbidities, frailty, and lower performance status are closely linked. Most first-line immunotherapy trials included patients with good performance status and without comorbidities, a significant predictor of survival [5]. Moreover, although there are validated tools, including the Charlson Comorbidity Index (CCI) and Simplified Comorbidity Score (SCS), the diversity and complex mechanisms of comorbidities make the subject even more challenging [4, 6, 7]. The subject is even more complicated by geriatric syndromes, including polypharmacy, potentially influencing survival outcomes through drug interactions, impairing immune responses, and effects on intracellular pathways, metabolism, inflammation, and tumor microenvironment [8–10]. Among these syndromes, sarcopenia is being increasingly recognized as a predictor of poor survival outcomes in patients receiving IO treatment [11, 12]. The effectiveness of IO treatment in elderly patients with multiple comorbidities appears to be a complex process that depends on numerous factors, many of which are poorly understood. In this study, we aimed to investigate the effect of comorbidities on IO treatment outcomes.

## Methods

This study was conducted in accordance with the “Declaration of Helsinki”. The institutional ethics committee approved the study protocol. All the patient data was recorded in an electronic database, and all the identities were blinded.

### Patient selection and outcomes

Patients with malignancies treated at our tertiary center were screened for eligibility. The data of patients who received immunotherapy in the locally advanced/metastatic setting and were over 18 years of age were retrieved from the hospital medical recording system. The patients’ data, such as age, gender, Eastern Cooperative Oncology Group (ECOG) performance status, pathologic diagnosis, treatment protocols, and CCI score were recorded at the time of diagnosis. The parameters required for calculating the CCI score were used in accordance with those in the original article [13]. The primary outcome was overall survival (OS).

### Statistical analysis

Descriptive statistics were given as mean and standard deviation or medians and interquartile range (IQR) for normally and non-normally distributed variables, respectively. The distribution of the variables was examined using visual (histogram and probability graphs) and analytical methods (Kolmogorov–Smirnov/Shapiro–Wilk tests). The Chi-Square test or Fisher’s exact test (when chi-square test assumptions do not hold due to low expected cell counts), where appropriate, was used to compare proportions in different groups. Independent samples *T*-test and Mann–Whitney *U* test were used to compare the means and medians, respectively. Maximally selected rank statistic was used to determine the cut-off value for CCI. OS was calculated from the initiation of systemic treatment to death. Survival analyses were investigated using Kaplan–Meier survival estimates and log-rank test. The possible factors identified with univariate analyses were further entered into Cox regression analysis to determine independent predictors of survival. A p-value of less than 0.05 was accepted as statistically significant. All statistical analyses were performed using IBM SPSS Statistics, version 26 (IBM Corp., Armonk, NY, USA).

## Results

A total number of 197 patients were included. The median age of the patients was 62 (IQR: 14) years. Non-small cell lung cancer was the most frequent diagnosis with a number of 67 (34%), followed by renal cell carcinoma (*n* = 46, 23.4%), and melanoma (*n* = 41, 20.8%). The most frequently used immunotherapy agent was nivolumab (*n* = 124, 62.9%), followed by pembrolizumab (*n* = 36, 18.3%), atezolizumab (*n* = 18, 9.1%), ipilimumab (*n* = 17, 8.6%), and nivolumab plus ipilimumab combination (*n* = 2, %1). Most patients were treated with immunotherapy as 2nd line or beyond (*n* = 150, 76.6%) in the metastatic setting. One-hundred-seven patients (54.9%) had metastatic disease at the time of diagnosis, with the lung being the most frequent site of metastasis (*n* = 100, 52.6%).

One-hundred-nineteen patients (60.4%) had no comorbidities. Fifty-three (26.9%) patients had one comorbidity, 19 (9.6%) patients had two comorbidities, and 6 (3%) patients had more than two comorbidities. The median survival times of these groups were 22.9 (95% Confidence Interval (CI): 11.8–34.1), 11.3 (95% CI 8.0–14.5), 9.3 (95% CI 5.5–13.1) and 1.4 (95% CI 0–9.6) months, respectively (*p* = 0.003, Supplementary Fig. 1).

Patients were grouped based on CCI scores: CCI-low (≤ 8) and CCI-high (> 8). Baseline characteristics were mostly similar in CCI-low and CCI-high groups. However, the number of patients with central nervous system metastases was significantly higher in the CCI-low group than in the CCI-high group (11.8% vs. 2.1%, *p* = 0.002). On the other hand, the median age at diagnosis was higher in the CCI-high group than in the CCI-low group (62 vs. 57 years, *p* < 0.001). Within the CCI-low group, 99 (88.3%) patients had no comorbidity, and 13 (11.7%) patients had at least one comorbidity. Patients’ characteristics are shown in Table 1.

**Table 1:** Baseline characteristics of the patients

	All patients		CCI ≤ 8		CCI > 8		*P*
	*n* = 197	(%)	*n* = 112	(%)	*n* = 85	(%)	
Age at initiation of IO, years, median, IQR	62 (14)		57 (10)		71 (10)		** < 0.001**
Sex							
Male	137	(69.5)	77	(39.1)	60	(30.5)	0.7
Female	60	(30.5)	35	(17.8)	25	(12.7)	
ECOG performance status							
0–1	167	(84.7)	100	(51.6)	67	(34.5)	0.2
≥ 2	27	(13.9)	11	(5.7)	16	(8.2)	
Tumor type							
Renal cell carcinoma	46	(23.4)	29	(14.7)	17	(8.6)	0.3
Melanoma	41	(20.8)	26	(13.2)	15	(7.6)	
Non-small cell lung cancer	67	(34)	37	(18.8)	30	(15.2)	
Small cell lung cancer	16	(8.1)	8	(4.1)	8	(4.1)	
Other*	27	(13.7)	12	(6.1)	15	(7.7)	
Immune checkpoint inhibitor							
Nivolumab	124	(62.9)	74	(37.6)	50	(25.4)	0.8
Pembrolizumab	36	(18.3)	18	(9.1)	18	(9.1)	
Ipilimumab	17	(8.6)	10	(5.1)	7	(3.6)	
Atezolizumab	18	(9.1)	9	(4.6)	9	(4.6)	
Ipilimumab + nivolumab	2	(1)	1	(0.5)	1	(0.5)	
IO treatment line							
1st	46	(23.4)	23	(11.7)	23	(11.7)	0.5
2nd and later	150	(76.6)	88	(44.9)	62	(31.6)	
Metastatic disease at the time of diagnosis	107	(54.9)	59	(30.3)	48	(24.6)	0.5
Sites of metastasis							
Lung	100	(52.6)	56	(29.5)	44	(23.2)	0.8
Bone	83	(42.8)	51	(26.3)	32	(16.5)	0.3
Liver	51	(26.3)	31	(16)	20	(10.3)	0.6
Central nervous system	27	(13.8)	23	(11.8)	4	(2.1)	**0.002**
Comorbidities							
Myocardial infraction	18	(9.1)	2	(1)	16	(8.1)	** < 0.01**
Congestive heart failure	6	(3)	0	(0)	6	(3)	** < 0.01**
Peripheral vascular disease	2	(1)	0	(0)	2	(1)	0.18**
Cerebrovascular event	6	(3)	1	(0.5)	5	(2.5)	0.08**
Dementia	3	(1.5)	0	(0)	3	(1.5)	0.07**
COPD	17	(8.9)	4	(2.1)	13	(6.8)	** < 0.01**
Connective tissue disease	8	(4.1)	1	(0.5)	7	(3.6)	0.02**
Peptic ulcer disease	1	(0.5)	0	(0)	1	(0.5)	0.43**
Liver disease							
Mild	4	(2)	2	(1)	2	(1)	1
Moderate to severe	0	(0)	0	(0)	0	(0)	
Diabetes mellitus							
Uncomplicated	23	(11.7)	5	(2.5)	18	(9.1)	** < 0.01**
End organ damage	21	(10.7)	0	(0)	21	(10.7)	
Hemiplegia	1	(0.5)	0	(0)	1	(0.5)	0.43**
Chronic kidney disease	0	(0)	0	(0)	0	(0)	–
Solid tumor	197	(100)	–	–	–	–	–
Leukemia	1	(0.5)	0	(0)	1	(0.5)	0.43**
Lymphoma	1	(0.5)	0	(0)	1	(0.5)	0.43**
AIDS	0	(0)	0	(0)	0	(0)	–

Statistically significant values are shown in bold

*COPD* chronic obstructive pulmonary disease, *IQR* interquartile range, *IO* immunotherapy

^*^Other types of cancer included head and neck, esophagus, gastric and colorectal cancer

^**^Fisher’s exact test

**Fig. 1:**
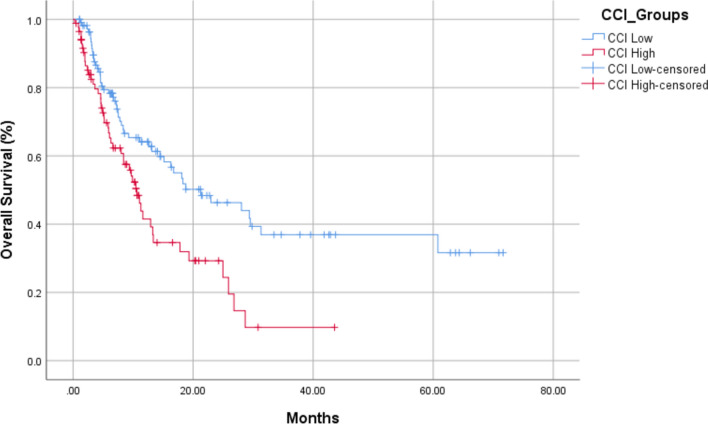
Overall Survival according to CCI groups

The median OS was shorter in the CCI-high group than in the CCI-low group (10.6 [95% CI 8.6–12.5] vs. 21.2 [95% CI 9.7–32.7] months, *p* = 0.002) (Fig. 1). Univariable analyses revealed advanced age (*p* = 0.012) and lower ECOG performance status (*p* = 0.001) to be significantly associated with poorer OS outcomes. Type of IO also affected OS, with anti-CTLA4 treatment associated with shorter OS (*p* = 0.024). In multivariable analysis, ECOG performance status of 2 or 3 (Hazard Ratio (HR): 2.17, 95% CI 1.25–3.75, *p* = 0.005), treatment with anti-CTLA4 (HR: 1.85, 95% CI: 1.07–3.20, p = 0.028), and higher CCI scores (HR: 1.97, 95% CI 1.3–3.0, *p* = 0.001) were associated with worse OS. Univariable and multivariable analyses of factors affecting OS are shown in Table 2.

**Table 2:** Univariable and multivariable analysis of factors affecting overall survival

	Univariable analysis		Multivariable analysis		
	Median OS (months)	*P*	Hazard Ratio	95% CI	*P*
Age					
< 65	19.3	**0.012**	1		
			1.25	0.75–2.0	0.386
≥ 65	10.6				
Sex					
Male	13.2	0.220			
Female	18.7				
ECOG performance status					
0–1	16.2	**0.001**	1		
≥ 2	4.6		2.25	1.32–3.83	**0.003**
Tumor type					
Renal cell carcinoma	21.2	0.114			
Melanoma	8.4				
Non-small cell lung cancer	18.7				
Small cell lung cancer	31.3				
Other*	28.6				
IO treatment line					
1st	15.1	0.824			
2nd and later	13.1				
Brain metastasis					
No	12.9	**0.048**	1		
Yes	29.5		0.66	0.33–1.31	0.243
Lung metastasis					
No	14.4	0.485			
Yes	13.3				
Liver metastasis					
No	17.8	0.160			
Yes	8.4				
Bone metastasis					
No	18.3	0.118			
Yes	12.9				
Lymph node metastasis					
No	19.3	0.495			
Yes	13.2				
IO plus chemotherapy					
No	13.2	0.176			
Yes	28.6				
IO toxicity					
No	12.9	0.082			
Yes	29.5				
Immune checkpoint inhibitor					
Anti-PD1	17.8	**0.024**	1		
Anti-PDL1	31.3		0.65	0.26–1.62	0.356
Anti-CTLA4	8.0				
			1.85	1.07–3.20	**0.028**
Combination	11.1		1.06	0.14–7.74	0.948
CCI group					
CCI low	21.2	**0.002**	1.98	1.30–3.00	**0.001**
CCI high	10.6				

Statistically significant values are shown in bold

*IO* immunotherapy, *CCI* Charlson Comorbidity Index, *OS* overall survival

^**^Other types of cancer included head and neck, esophagus, gastric and colorectal cancer

## Discussion

Our study highlights that the comorbidity burden significantly impacts survival outcomes in advanced cancer patients receiving IO. Specifically, patients with higher CCI scores had notably shorter OS compared to those with lower CCI scores. Additionally, ECOG performance status was a key independent predictor, with poorer status associated with reduced OS. These results confirm that both comorbidity burden and performance status are critical factors influencing IO outcomes, highlighting the need for a comprehensive assessment of these parameters before initiating treatment.

The relationship between comorbidities and immune mechanisms has been a subject of research for a long time. Information is accumulating, especially on the relationship between chronic diseases and inflammation [14, 15]. It is known that systemic proinflammatory processes can cause microvascular endothelial changes and tissue damage through various intracellular mechanisms [16]. It is also known that conditions such as arterial hypertension, diabetes mellitus, and hyperlipidemia trigger inflammation and increase the risk of developing many vascular and degenerative pathologies, including the central nervous system [17, 18]. This inflammatory milieu caused by chronic diseases possibly influences the IO response, as well as age-related immune deterioration [19]. The mechanism of this phenomenon, immunosenescence, includes having fewer B and T cells, increased immune-suppressive cells and alterations on other cells that is needed for T cell activity [20–24], therefore deteriorating IO response rates.

It is well established that ECOG performance status is a strong predictor of survival [5]. While advanced age is a risk factor for comorbidities and worse performance status, there is growing evidence that relying solely on age to make treatment decisions may be misleading. In a study evaluating non-small cell lung cancer patients over the age of eighty, it was shown that the rate of systemic treatment was significantly lower in patients with advanced age and high comorbidity scores, whereas the survival of patients receiving IO was significantly higher [25]. The findings of this study suggest that even patients with multiple comorbidities may benefit from IO treatment. Our study revealed that not age, but ECOG performance status and CCI scores were independent factors influencing OS. These findings indicate that advanced age alone may not be a determinant for survival in the absence of significant comorbidities or poor performance status. Another study examining the use of IO and comorbidity rates in patients with metastatic small-cell lung cancer showed that the rate of IO use was significantly lower in patients with a comorbidity score of 2 and above [26]. As outlined above, elderly patients with comorbidities, especially patients with poorer performance status, are largely underrepresented in the studies, leading to conflicting results. In a meta-analysis evaluating six randomized controlled trials and 32 non-randomized controlled trials in patients with advanced urothelial cancer, poorer performance status in patients receiving IO in non-randomized controlled trials was shown to be associated with poor survival outcomes.

Apart from its effect on performance status, there are studies reporting that comorbidities may have an impact on treatment responses. One study demonstrated that lower CCI and SCS scores were associated with better disease control rates and progression-free survival [3]. Another study demonstrated that inflammatory markers, CCI scores and immune adverse events are associated with mortality [4]. In a study that included 671 patients, findings suggested that cardiovascular disease may predict shorter OS in patients with immune-related adverse events [6]. However, in a study including 51 patients over the age of 75 who had advanced-stage non-small cell lung cancer and received immunotherapy, no association was found between CCI scores and prognosis [27]. In our study, while the CCI score was an independent prognostic factor for OS, multivariate analyses revealed no significant effect on PFS. These conflicting results further indicate that elderly patients with multiple comorbidities constitute a heterogeneous group, with various factors affecting survival and IO treatment responses. There are studies in the literature that evaluated inflammatory markers in combination with comorbidities and their impact on survival [28]. Considering the important role of inflammation in the pathogenesis of comorbidities, these findings suggest that they may also influence the treatment response and adverse effect profile through complex immune-mediated mechanisms. In our study, significant immune-mediated adverse events did not differ between CCI groups, but there are also reports indicating that comorbidities may be associated with the frequency of adverse events [29]. Given the complexity of the situation and conflicting results, further studies are warranted to elucidate the effect of different comorbidities on IO treatment responses.

Anti-CTLA4 treatment was associated with a poorer survival outcome, however, this finding may be misleading due to the small number of patients, which may be due to the advancements in targeted therapies and IO combination strategies. The finding that the presence of brain metastasis being associated with favorable outcomes is somewhat unexpected. However, in multivariate analysis, no significant effect on survival was demonstrated. As previously mentioned, the number of patients with brain metastases was quite small and these patients were significantly younger, and, therefore, expected to have a more favorable performance status and fewer comorbidities, which would explain the difference in survival, since the presence of brain metastasis could not be demonstrated as an independent risk factor.

This study has several limitations inherent to its retrospective design, which may introduce biases such as incomplete or missing data. Additionally, our analysis did not account for certain geriatric syndromes like polypharmacy and sarcopenia, which could have influenced treatment outcomes and patient survival.

We also did not include inflammatory markers or detailed data on potential drug interactions, which are important factors when evaluating the immune response and treatment efficacy. The relatively small sample size, particularly in subgroups such as those receiving anti-CTLA4 therapy and patients with brain metastases, may reduce statistical power and limit the generalizability of our findings. Therefore, prospective studies with larger patient counts are necessary to confirm our findings’ generalizability.

Despite these limitations, our study has several strengths. It is one of the few to evaluate the impact of comorbidity burden and performance status on survival outcomes in a diverse cohort of cancer patients undergoing immunotherapy. By using a well-established comorbidity assessment tool (CCI), we provided an objective and reliable evaluation of comorbidity burden as a predictor of outcomes. Furthermore, the inclusion of a substantial number of patients from various cancer types enhances the robustness of our findings and their relevance to real-world clinical practice. Our study also contributes to the growing body of evidence supporting the need for comprehensive assessment in personalized cancer treatment, offering valuable insights for optimizing therapeutic strategies in patients with complex clinical profiles.

Our study demonstrates that comorbidity burden, as assessed by the CCI, and ECOG performance status are independent predictors of survival outcomes in advanced cancer patients receiving immune checkpoint inhibitors. These findings underscore the importance of a comprehensive evaluation of comorbidities and functional status before initiating immunotherapy, especially in elderly patients or those with complex clinical profiles. While our results highlight the need for personalized treatment approaches, further prospective studies are warranted to refine the CCI and integrate additional prognostic markers such as inflammatory indices and geriatric syndromes like sarcopenia.

## Supplementary Information

Below is the link to the electronic supplementary material.

Supplementary file1 (DOCX 39 KB)

## Data Availability

The data that support the findings of this study are available on request from the corresponding author, YÜ.
