# Supplementary figures and images for: Impact of comorbidity on survival in cancer patients receiving immune checkpoint inhibitors
Source: Clin Transl Oncol. 2025 Jan 28;27(7):3175–82. doi: 10.1007/s12094-025-03848-7 (PMC12179231; doi:10.1007/s12094-025-03848-7)

Supplementary Figure 1: Overall Survival according to number of comorbidities


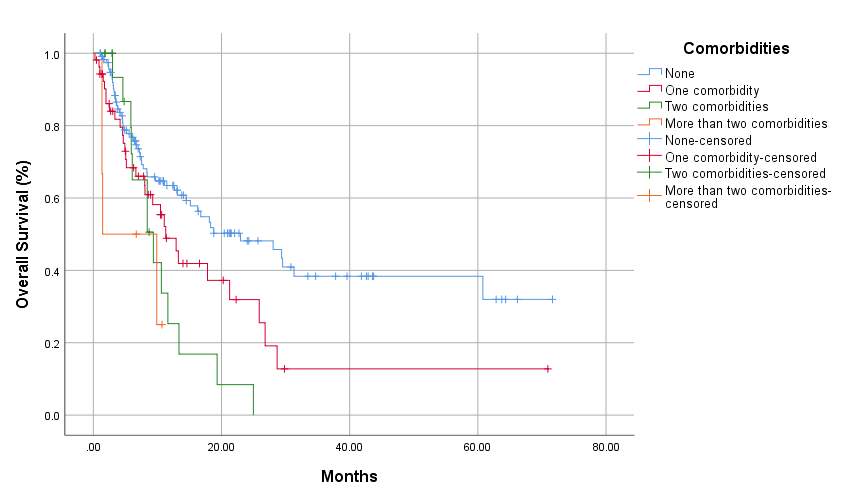

Supplement: Supplementary file 1 — Supplementary file1 (DOCX 39 KB) [file 12094_2025_3848_MOESM1_ESM.docx]
